# Case Report: MSS colorectal extrahepatic (non-liver) metastases as the dominant population for immunotherapy combined with multi-target tyrosine kinase inhibitors

**DOI:** 10.3389/fonc.2023.1091669

**Published:** 2023-03-10

**Authors:** Jiayin Liu, Dan Li, Jing Han, Yin Zhang, Xue Zhang, Zhisong Fan, Li Feng, Long Wang, Hui Jin, Jing Zuo, Yudong Wang

**Affiliations:** ^1^ Department of Medical Oncology, The Fourth Hospital of Hebei Medical University and Hebei Provincial Tumor Hospital, Shijiazhuang, China; ^2^ Inspection Department, Shijiazhuang Institue for Food and Drug Control, Shijiazhuang, Hebei, China

**Keywords:** MSS, extrahepatic (non-liver) metastases, dominant population, immunotherapy combined, multi-target tyrosine kinase inhibitors

## Abstract

**Background:**

The microsatellite stability(MSS) subtype of Colorectal Cancer(CRC) represents approximately 95% of mCRC cases. Immunotherapy was not as encouraging as the data for MSS mCRC cancer. We report the treatment of a series of patients with extrahepatic metastasis of MSS colorectal cancer, which can provide reference and guidance for the treatment of non-hepatic metastasis of MSS colorectal.

**Case presentation:**

This report describes 8 typical cases of successful MSS treatment with lung metastases of CRC. We systematically reviewed the clinical data and detailed medical history of one of these patients with extrahepatic metastasis from MSS colorectal cancer, and reviewed the literature to analyze and discuss the related epidemiological features, mechanisms and recent research findings of the special subgroup of the population.

**Conclusions:**

Although MSS colon rectal cancer is still known as a cold tumor in the industry, immunotherapy combined with multi-targeted anti-vascular tyrosine kinase inhibitors had brought clinical benefits to patients with non-hepatic metastases from MSS colorectal cancer.

## Introduction

According to the most recent cancer report published by the National Cancer Center of China (1), colorectal cancer (CRC) has become the second most common malignant tumor after lung cancer, with about 408,000 new cases. The incidence and mortality of CRC ranks among the top second malignant tumors and has become one of the main cancers that can endanger life and health.

About 20% of patients present distant metastases at the time of initial diagnosis, of which the liver and lung are the most representative sites. The lung is the organ most likely to metastasize after the liver, and 5–15% of patients will eventually develop lung metastases. Treatment of lung metastases has become an integral part of the comprehensive treatment of CRC. The general treatment of CRC multiple pulmonary metastases mainly involves drug therapy, including systemic chemotherapy and targeted drug therapy. After drug treatment, some patients may achieve a reduction in the size of original unresectable lesions and achieve resectable conditions. Patients with resectable metastases have a 5-year survival rate of approximately 30–40%.

The MisMatch Repair-deficient(dMMR)/microsatellite instability-high(MSI-H) subtype of CRC represents approximately 15% of all cases and 5% of mCRC cases. Due to the high mutation rate of dMMR/MSI-H, tumors are highly immunogenic, enabling them to activate the antitumor response of the immune system. dMMR/MSI-H patients have been reported to be more responsive to immune checkpoint inhibitor (ICI)-based immunotherapy. In the KEYNOTE-016 trial, investigators found that multiple tumors in dMMR benefited from pembrolizumab immunotherapy, and in mCRC, pembrolizumab monotherapy resulted in an objective response rate 57% in cases of dMMR, while the ORR was 0 in patients with pMMR (2).

Furthermore, KEYNOTE-164 and 158 studies confirmed that pembrolizumab produced an ORR of 33% and a long-term survival benefit in previously treated patients with advanced MSI-H CRC (3, 4). Based on the excellent results of five studies including KEYNOTE-016, 164, and 158 trials, the FDA approved pembrolizumab in 2017 for the treatment of patients with solid tumors with MSI-H/dMMR, including mCRC. Although the above studies affirmed the benefit of immunotherapy in patients with MSI-H/dMMR mCRC, it is not recommended as first-line treatment of advanced patients.

For 95% of MSS bowel cancer patients, immunotherapy was not as encouraging therapy as the data for advanced MSI-H/dMMR bowel cancer. In contrast, MSS bowel cancer is still called a cold tumor in the industry, and single-drug immunotherapy has little effect on advanced bowel cancer. Basic research suggests that the level of lymphocytes infiltrating the MSS tumor microenvironment is low, and the immune response is weak. The KEYNOTE016 phase II study and the KEYNOTE-028 IB study also showed that patients with normal mismatch repair (pMMR) CRC did not respond to pembrolizumab therapy. How to change the immune microenvironment and how to turn a cold tumor into a hot tumor has become the biggest bottleneck in the immunotherapy of advanced CRC. However, recent studies related to combination therapy have raised the possibility of improving the efficacy of immunotherapy in this population. The combination of immunotherapy and small-molecule antiangiogenic targeted therapy has made significant progress. This report describes a typical case series of successful MSS treatment with lung metastases of CRC.

## Case presentation

A 60-year-old female was referred to our hospital in June 2016 for increased stool frequency for more than six months. Her mother died of “rectal cancer”. Colonoscopy showed ulcerated neoplasia of the rectum 8-13 cm from the anal verge, invading nearly half of the circumference. Pathology on bite-examination was adenocarcinoma of the rectum. The abdominal MRI showed a rectal wall mass consistent with rectal cancer, with multiple enlarged lymph nodes around the rectal mesentery and superior rectal artery; the rest of the pelvis was not abnormal. The patient’s imaging stage was cT3N2M0.

**Figure 1:**
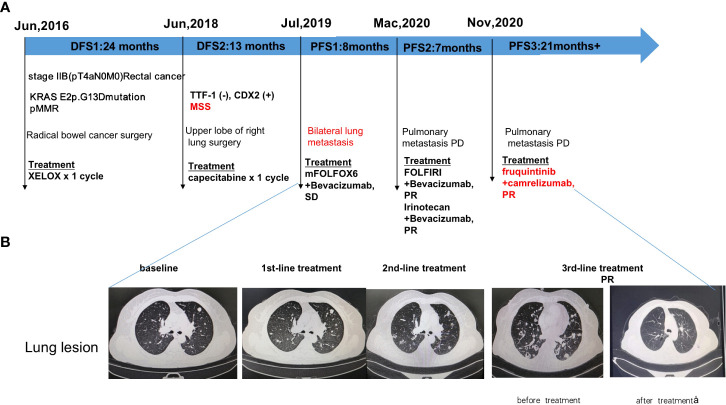
Diagram of patient’s treatment history and clinical course. **(A)** Timeline of treatment; **(B)** Imaging changes before and after treatment. PR: partial response; SD: stable disease; PFS: progression-free survival.

Radical bowel cancer surgery: In June 2016, the patient underwent a radical Dixon operation for rectal cancer. The postoperative pathology showed grade II adenocarcinoma invading the mesentery, with no clear choroidal aneurysm embolus or nerve invasion. Lymph nodes: peri-intestinal 0/10, mesenteric 0/4, mesenteric root 0/4 metastases. The patient was diagnosed with stage IIB (pT4aN0M0) adenocarcinoma. Genetic testing suggested KRAS E2p.G13D mutation (**Figure 1A**). In July 2016, the patient started postoperative treatment with the XELOX protocol for one cycle. Patients are not tolerated due to adverse reactions and are reviewed regularly (**Figure 1A**).

In June 2018, CT scan suggests nodule in upper lobe of right lung, metastasis? Not excluding primary lung cancer. Disease-free survival after bowel cancer surgery was 2 years (DFS).

Upper lobe of right lung surgery: In June 2018, the patient underwent right upper lung lobectomy. Immunohistochemistry suggested TTF-1 (-), CDX2 (+), Napsin A (-), PDL1 (DAKO 22C3) (0% positive), PD-L1 (VENTANA SP263) (0% positive), ALK Negative (-), BRAF (-), MLHI1 (+), PMS2 (+), MSH2 (+), MSH6 (+). Pathology suggests adenocarcinoma, considered of intestinal origin. In July 2018, the patient was given capecitabine chemotherapy for one cycle. Patients are not tolerated due to adverse reactions and are reviewed regularly **(** **Figure 1A** **)**. Disease-free survival after excision of lung metastases was 13 months(DFS).

1^st^-line treatment: In July 2019, CT scan suggested multiple nodules in both lungs and metastases were considered. A whole-body bone scanning indicated bone metastases. The patient started 1st-line treatment with mFOLFOX6+bevacizumab, lasting for seven cycles, and the disease was stable which achieved stable disease (SD) of the lung lesion (**Figure 1A**). Subsequently, the patient was not diagnosed and treated as planned due to the impact of the epidemic and remained on SD until March 2020 with a PFS of 8 months.

2^nd^-line treatment:In March 2020, CT scan showed enlarged multiple metastases in both lungs, the patient developed a progressive disease (PD). The patient started 2^nd^-line treatment with FOLFIRI+Bevacizumab, lasting for eight cycles, and the disease was stable which yielded a partial response partial response (PR) of the lung lesion (**Figure 1A**). 2^nd^-line maintenance therapy was given with irinotecan and bevacizumab, lasting for two cycles and until November 2020 with a PFS of 7 months.

3^rd^-line treatment : Starting from November 2020, patients received a combinatorial treatment with furoquinitinib (5mg d1-14 q21d), camrelizumab (200mg d1 q21d),lasting 23 cycles so far. The patient received 3^rd^-line treatment achieved maintain partial response (PR)(**Figure 1B**).

## Discussion

Currently, the lungs are the second most common site of CRC metastases after the liver. As rectal cancer patients are prone to lung metastases (5) and the proportion of rectal cancer cases in China (nearly 50%) is significantly higher than that reported in western countries (around 30%) (6), the diagnosis and treatment of lung metastases in CRC indicated more significant clinical problems in China.

MSI-H patients account for only 5% of advanced CRC patients, and the remaining 95% of CRC patients are generally genotyped as MSS. To date, the efficacy of immunotherapy among mCRC patients with MSS has not been satisfactory, but significant research is currently underway (7).

Recent research has focused on improving clinical response rates and generalizing these treatments to all CRCs (8). Cancer patients with liver metastases demonstrate significantly worse outcomes than those without liver metastases when treated with anti PD-1 immunotherapy. The research report that patients with both hepatic and extrahepatic metastasis showed more favorable survival and higher response to dual ICB than those with hepatic metastasis only (9). Cancer cells invading the liver may trigger liver specific tolerance mechanisms that reduce systemic antitumor immunity and cancer immunotherapy efficacy. Clinical data reveals that liver metastasis patients are less responsive to treatment with anti PD-1 antibodies than patients without liver metastases, which is confirmed from basic research studies (10, 11). The liver facilitates distant immune suppression of tumor antigens independently of tumor burden. Tregs undergo specific priming in the presence of liver tumor, and enhanced Tregs can modify tumor-antigen specific MDSCs that migrate to distant sites, ultimately suppressing antigen-specific CD8+ T cell activation *via* clonal anergy. Furthermore, liver metastasis is correlated with a decreased efficacy of immunotherapy in cancer patients. Liver metastases can attract CD8^+^ T cells from systemic circulation. Within the liver, activated antigen-specific FasL^+^CD8^+^ T cells undergo apoptosis following their interaction with FasL^+^CD11b^+^F4/80^+^ monocyte-derived macrophages.

NCCN guidelines and CSCO guidelines recommend regorafenib or fruquintinib or TAS-102 as limited treatment options, but the survival benefits after the third line is not ideal. The recently published REGONIVO study (12), which explored the combination of immunotherapy (nivolumab) with an antiangiogenic targeted therapy (regorafenib), showed that the combined regimen achieved an ORR of 33% in MSS/pMMR mCRC. The researchers believed that the efficacy of combination therapy indicates that anti-angiogenesis therapy may improve the immune status of the tumor microenvironment and relieve the immunosuppressive effect, which enhances outcomes of immunotherapy. In addition, patients who benefited from this combined treatment were all male and had lung metastases, which may have implications for the selection of the treatment population.

One study (13) suggests that the combination of fruquintinib and sintilimab reduced angiogenesis and reprogramed the liver vascular structure, enhanced infiltration of CD8+ T cells (p<0.05), CD8+ TNFa+ (p<0.05) T cells, and CD8+ IFNg+ (p<0.05) T cells and reduced the ratios of MDSCs and macrophages in mice. Furthermore, fruquintinib can correct the immune escape microenvironment of tumor cells, mainly by inhibiting PD-L1 expression, inhibiting tumor release of inflammatory factors and immunosuppressive factors such as IL6/IL-10/VEGFR, and inhibiting bone marrow-derived suppressor cells. In turn, the secretion of T-reg cells is inhibited and the microenvironment is conducive to synergistic immunotherapy.

Based on the above clinical studies, we select the appropriate patients in the clinic. From the eight patients selected, the combination of immunotherapy and targeted therapy in the treatment could benefit (**Table 1**). These data have also been confirmed in our clinical practice.

**Table 1:** Demographic features, clinical characteristics, and therapeutic regimens.

Items	Patient 1	Patient 2	Patient 3	Patient 4	Patient 5	Patient 6	Patient 7	Patient 8
**Gender**	Female	Female	Female	Female	Male	Female	Female	Female
**Age (years)**	65	60	68	76	54	57	55	61
**location**	Colon Cancer	Colon Cancer	Colon Cancer	Rectal Cancer	Colon Cancer	Colon Cancer	Colon Cancer	Rectal Cancer
**PS**	1	1	1	1	1	1	1	1
**Treatment** **Lines**	3 lines	3 lines	3 lines	3 lines	3 lines	3 lines	3 lines	3 lines
**Metastasis** **Site**	Lung	Lung, bone	Lung	Lung	Peritoneal	Peritoneal	Lung	Lung
**KRAS** **Status**	mutation	mutation	Wild	mutation	mutation	mutation	mutation	mutation
**MMR/MSS**	pMMR	MSS	pMMR	pMMR	pMMR	pMMR	MSS	pMMR
**1 Lines**	XELOX+ Bevacizumab	XELOX+ Bevacizumab	mFOLFOX6+ cetuximab	X+Bevacizumab	XELOX+ Bevacizumab	XELOX+ Bevacizumab	mFOLFOX6+ Bevacizumab	mFOLFOX6+Bevacizumab
**2 Lines**	FOLFIRI+Bevacizumab	FOLFIRI+Bevacizumab	XELIRI+cetuximab	FOLFIRI+Bevacizumab	FOLFIRI+Bevacizumab	FOLFIRI+Bevacizumab	FOLFIRI+Bevacizumab	FOLFIRI+Bevacizumab
**3 lines**	Fruquintinib camrelizumab	Fruquintinib camrelizumab	Fruquintinib camrelizumab	Fruquintinib camrelizumab	Fruquintinib camrelizumab	Fruquintinib camrelizumab	Fruquintinib camrelizumab	Fruquintinib camrelizumab
**Circles**	12	23	15	6	7	13	14	16
**Best Response**	PR	PR	PR	PR	SD	SD	PR	PR
**PFS**	12M+	21M+	17M+	7M+	7M+	14M+	15M+	18M+

Description: The deadline for statistics is August 2022.

Patient 2 in the table is the case presentation patient in the article

Meanwhile, we analyzed the hematological data of 8 patients before and after treatment, including blood routine, biochemical indexes (**Table 2**) and tumor markers. We analyzed the changes of tumor markers (including CEA, CA199 and CA724) before and after treatment in 8 patients (**Supplementary Material**). It can be seen that there is a downward trend in tumor markers before and after treatment.

**Table 2:** Baseline blood routine and biochemical indicators.

Items	Patient 1	Patient 2	Patient 3	Patient 4	Patient 5	Patient 6	Patient 7	Patient 8
Gender	Female	Female	Female	Female	Male	Female	Female	Female
**Age (years)**	65	60	68	76	54	57	55	61
**3 lines Start time**	2021.8	2020.11	2021.3	2022.1	2022.1	2021.6	2021.5	2021.2
**WBC X10^9/L**	4.26	8.37	4.05	6.25	8.37	3.64	4.41	4.55
**RBC X10^12/L**	3.86	4.6	3.84	4.16	4.63	4.28	3.36	3.89
**HGB g/L**	125.9	145.8	137	136	139	135	109	115
**NE X10^9/L**	2.71	4.5	2.18	2.93	4.52	1.79	2.75	2.95
**PLT X10^9/L**	154	244	165	124	168	259	259	230
**LY X10^9/L**	1.11	2.75	1.5	2.59	2.19	1.24	1.29	1.93
**NLR(NE/LY)**	2.44	1.63	1.45	1.13	2.06	1.44	2.13	1.52
**PLR(PLT/LY)**	138.73	88.72	110	109.7	76.71	215.8	200.77	119.17
**ALT U/L**	17.1	20.4	21.3	24	23.6	21.4	17.7	23
**AST U/L**	22	22.7	24.9	28	24	23.2	21.3	25
**ALB g/L**	41.8	39.5	42.5	39.8	40.1	43.4	42.2	39
**CREA umol/L**	58.7	54.5	48.2	80	83.7	50.5	44	51
**LDH U/L**	188	155	176	146.5	236	164	143	156

## Conclusions

Sample population selection is also critical when formulating treatment regimens. Our case suggests that MSS colorectal extrahepatic (non-liver) metastases as the dominant population for immunotherapy combined with multi-target tyrosine kinase inhibitors. Therefore, the combined antivascular immunotherapy is promising, and we can look forward to a new treatment landscape for MSS CRC in the future.

## Data availability statement

The original contributions presented in the study are included in the article/**Supplementary Material**. Further inquiries can be directed to the corresponding author.

## Ethics statement

The authors are accountable for all aspects of the work in ensuring that questions related to the accuracy or integrity of any part of the work are appropriately investigated and resolved. All procedures performed in this study were in accordance with the ethical standards of the institutional and/or national research committee(s) and with the Helsinki Declaration (as revised in 2013). This study was conducted with Fourth Hospital of Hebei Medical University Research Ethics Board approval (2021KS005). Written informed consent was obtained from the patient’s family for publication of this case report and accompanying images. A copy of the written consent is available for review by the editorial office of this journal.

## Author contributions

All authors made a significant contribution to the work reported, whether that is in the conception, study design, execution, acquisition of data, analysis and interpretation, or in all these areas; took part in drafting, revising or critically reviewing the article; gave final approval of the version to be published; have agreed on the journal to which the article has been submitted; and agree to be accountable for all aspects of the work.
