# Supplementary figures and images for: Case Report: MSS colorectal extrahepatic (non-liver) metastases as the dominant population for immunotherapy combined with multi-target tyrosine kinase inhibitors
Source: Front Oncol. 2023 Mar 10;13:1091669. doi: 10.3389/fonc.2023.1091669 (PMC10036805; doi:10.3389/fonc.2023.1091669)

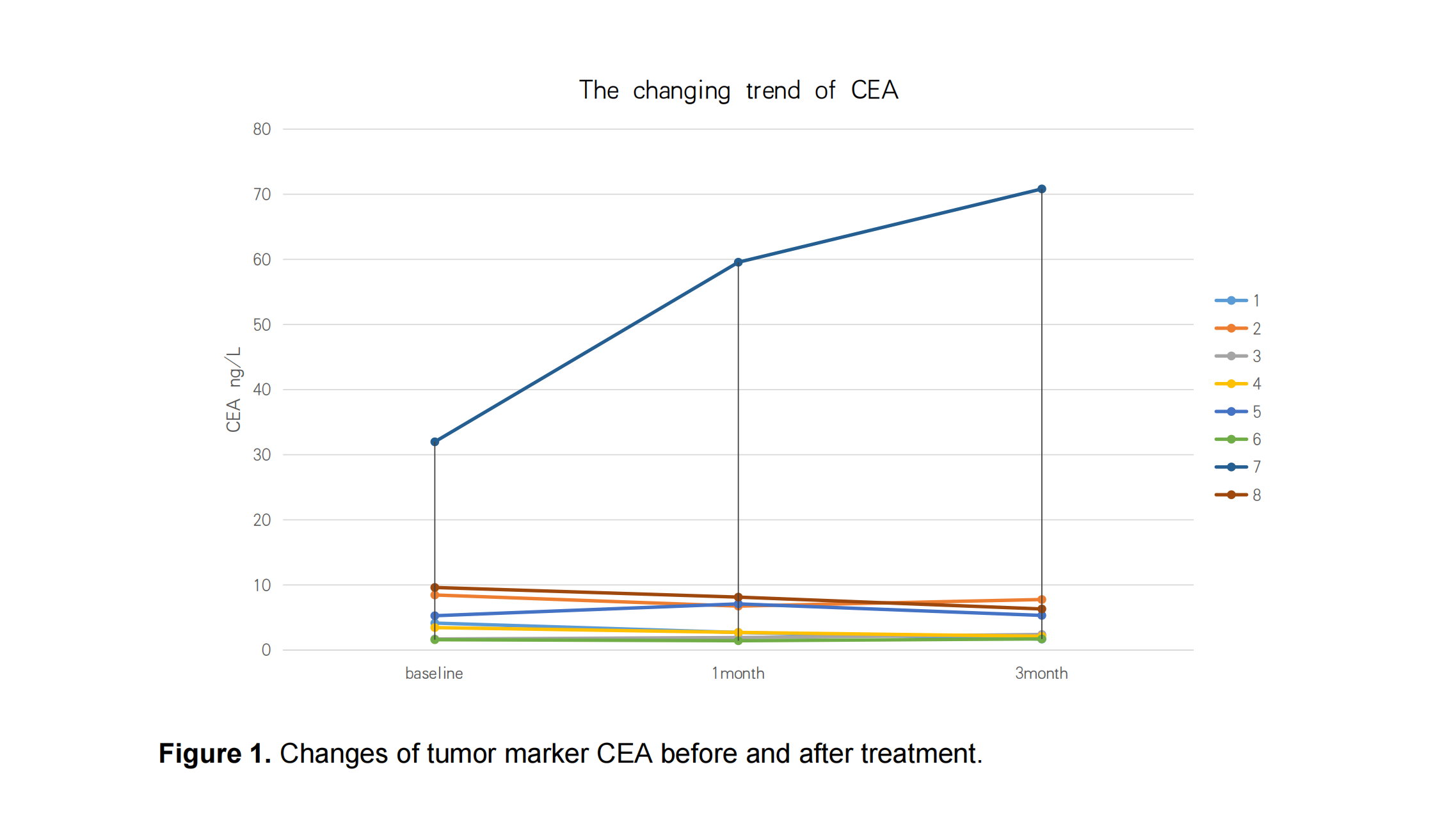

Supplement: Supplementary file 1 [file Image_1.png]

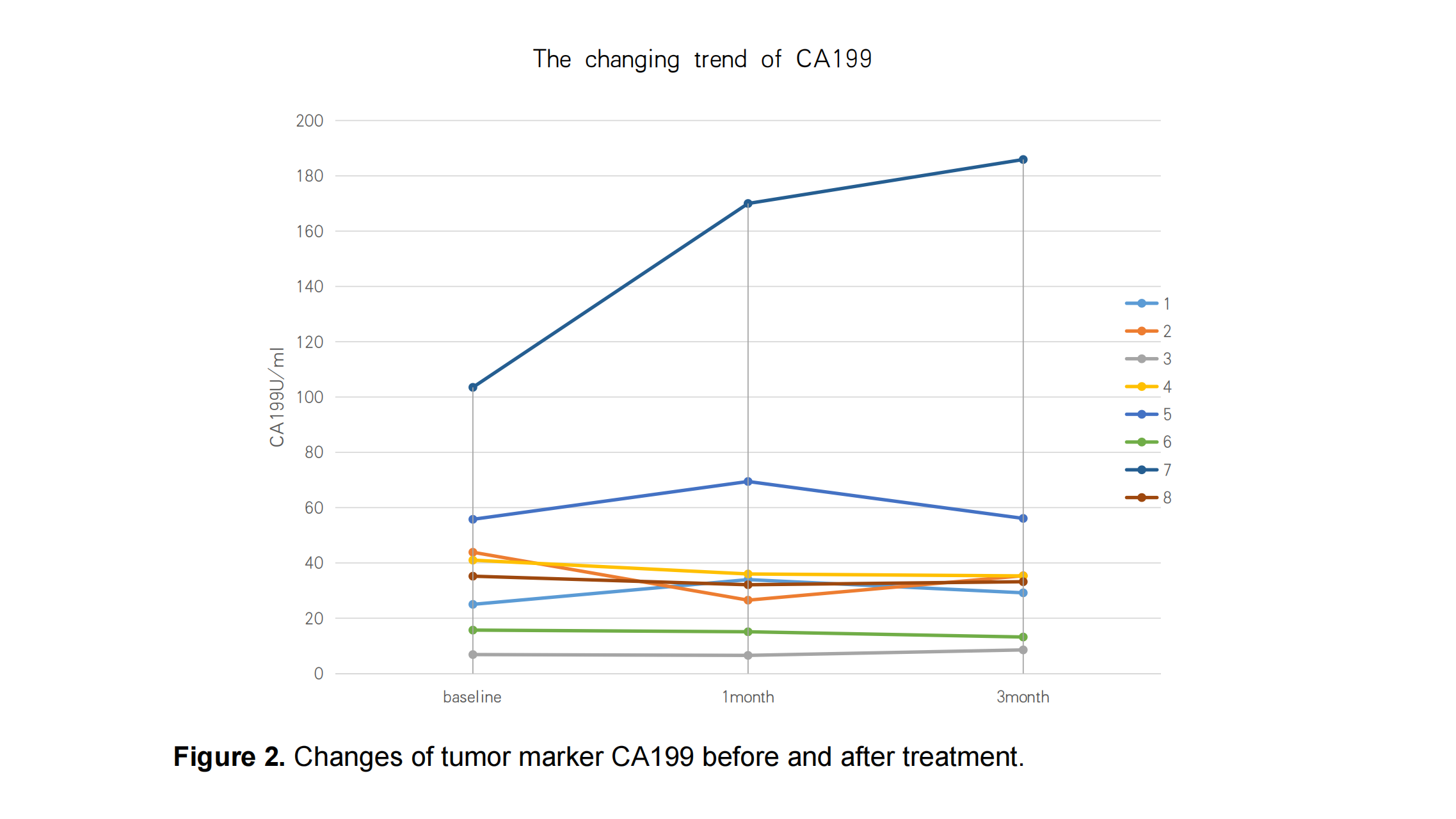

Supplement: Supplementary file 2 [file Image_2.png]

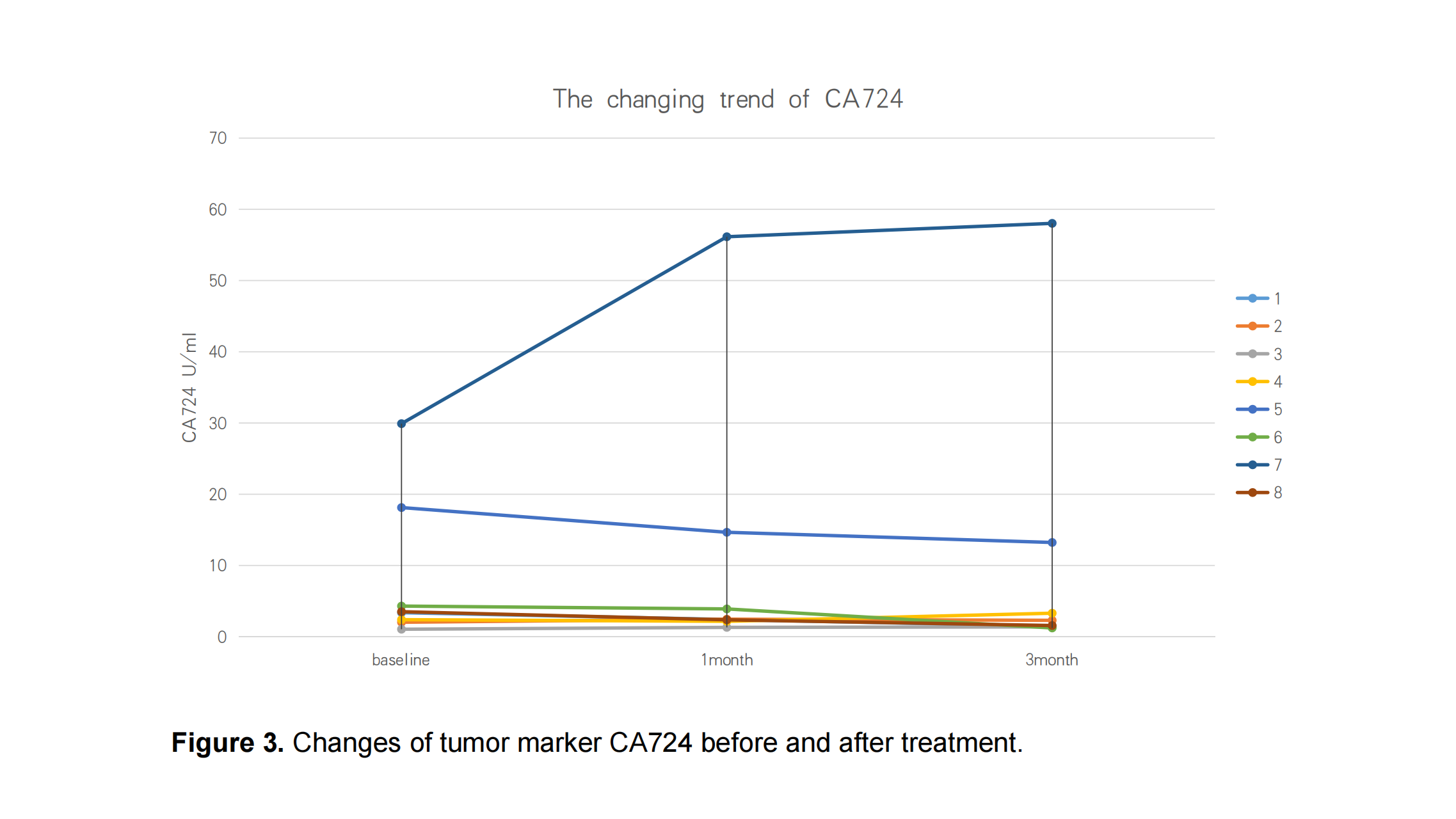

Supplement: Supplementary file 3 [file Image_3.png]
